# The role of temporal coherence and temporal predictability in the build-up of auditory grouping

**DOI:** 10.1038/s41598-022-18583-0

**Published:** 2022-08-25

**Authors:** Joseph Sollini, Katarina C. Poole, Dominic Blauth-Muszkowski, Jennifer K. Bizley

**Affiliations:** 1 Hearing Sciences, Mental Health and Clinical Neurosciences, School of Medicine, University of Nottingham, Nottingham, England, UK; 2 The Ear Institute, University College London, London, England, UK

**Keywords:** Auditory system, Sensory processing

## Abstract

The cochlea decomposes sounds into separate frequency channels, from which the auditory brain must reconstruct the auditory scene. To do this the auditory system must make decisions about which frequency information should be grouped together, and which should remain distinct. Two key cues for grouping are temporal coherence, resulting from coherent changes in power across frequency, and temporal predictability, resulting from regular or predictable changes over time. To test how these cues contribute to the construction of a sound scene we present listeners with a range of precursor sounds, which act to prime the auditory system by providing information about each sounds structure, followed by a fixed masker in which participants were required to detect the presence of an embedded tone. By manipulating temporal coherence and/or temporal predictability in the precursor we assess how prior sound exposure influences subsequent auditory grouping. In Experiment 1, we measure the contribution of temporal predictability by presenting temporally regular or jittered precursors, and temporal coherence by using either narrow or broadband sounds, demonstrating that both independently contribute to masking/unmasking. In Experiment 2, we measure the relative impact of temporal coherence and temporal predictability and ask whether the influence of each in the precursor signifies an enhancement or interference of unmasking. We observed that interfering precursors produced the largest changes to thresholds.

## Introduction

It is often hard to fathom how the auditory system is able to turn a simple two-dimensional signal, consisting of variations in pressure over time, into the complex sound scene we perceive. The auditory system uses cues to group together information arising from separate sources, or ‘objects’^1–4^ and segregate information arising from distinct sources^5^. Here we focus on two important cues and their role in establishing auditory grouping, temporal coherence and temporal predictability. Sound elements from a common source will often fluctuate in power simultaneously. These synchronous changes in amplitude across-frequency serve as a critical grouping cue, useful for sound detection^6,7^ and speech processing^8^, and can be referred to as either comodulation^9^ or temporal coherence^4^ (Note: as the amplitude fluctuates the availability of other cues will become available/unavailable and temporal coherence refers to the fluctuation in these cues over time also). A sound’s statistics can be stationary over time (for example a pure tone of fixed frequency) or vary over a defined range, such as natural sound textures^10^. Reliability in these statistics over time also improves the segregation of auditory objects^11–13^. The degree of reliability of these statistics, the temporal predictability, can be varied on a continuum from 100% predictable, often called “regular” where the statistics are identical over time^14^, to 0% predictable, also called random.

The auditory system is excellent at gathering information over time to improve perception, this process of accumulating knowledge about a sound is often referred to as the “build-up” period. Build-up has been observed in a range of seemingly disparate auditory behaviours, such as listening to speech-in-noise^15^, speech in speech^11^, auditory stream formation^16^, the discrimination of sound textures^10^, comodulation masking release^17^, the precedence effect^12^ and transitions to new spectrotemporal sound statistics^18^. Generally speaking, the longer the build-up period, the greater the behavioural benefit^10,12,19–21^, although, this benefit will asymptote and can subsequently decline due to inattention^22^. The optimal build-up period can be thought of as the shortest duration of time over which performance begins to asymptote, i.e. when additional build-up offers very little behavioural advantage. The optimal build-up period varies depending on the task, but appears to be between 0.4 and 2.5 s^10,12,15,19^, though further modest gains can be made for more than a minute^16,23^. In the case of “predictable” sounds, i.e. repeating spectrotemporal structure, this variation can be explained by the duration or period of the sound statistics themselves, i.e. the longer a repeating sequence the more time is required to learn that sequence^18^.

Once an object has been built-up it can take several seconds for its influence to decay^16,24^ unless object formation is reset by abrupt changes in an ongoing sound, e.g. a frequency, level or spatial deviation in one of the objects^23,25–28^. Overall, the existing literature suggests that build-up is a high-level evidence accumulation process^21^ that influences auditory processing. It can do this by either adapting to or enhancing the important features within an object and operates on a range of grouping cues. Establishing the structure in an auditory scene then serves a range of core auditory abilities, such as segregation^11,16^, detection^13,29^ or discrimination^10^. It is not clear whether build-up is one general process or whether independent build-up mechanisms subserve different grouping cues. To better understand this, it is necessary to investigate interactions between different grouping cues in order to measure their influence upon one another.

Here, we investigate how temporal predictability and temporal coherence interact with one another by manipulating the cues available during the build-up period. Given the reasonably long-lasting effects of build-up (up to 1 s) these interactions should have important consequences on the perception of subsequent sounds. Indeed, grouping processes have been shown to effect auditory stream segregation^16,30^ and also sound detection^31–33^. We chose to employ a simple tone in background sound detection task in which the likelihood of detecting the tone was influenced by the ability of listeners to successfully segregate the tone and masker. This provides an indirect but objective measure of how the listener is grouping the sound mixture. Here, increases in tone detection threshold indicate an impaired ability to represent both sounds (masker and tone) separately, while decreases in detection threshold suggest that the masker and tone are perceptually better segregated. We sought to promote perceptual segregation with the use of either temporal coherence and/or temporal predictability as a grouping cue during the build-up period. By varying the background sound statistics during build-up, while providing both cues later in the background (when the tone was presented) we were able to assess how these statistics influenced subsequent grouping. Using this approach, we were able to show that manipulating temporal predictability and coherence in the build-up can significantly affect the auditory system’s ability to make use of these grouping cues. Our results demonstrate the importance of the build-up period on organising an auditory scene and thus the subsequent perception of auditory objects.

## Materials and methods

### Participants

All participants were aged between 19 and 25 and none reported long-term or current problems with their hearing. Experimental procedures were carried out in accordance with the protocols approved by the research ethics committee of University College London (reference 3866/002) and written informed consent was provided by each participant. Participants were given a short training session (usually lasting 15–20 min) to familiarise themselves with the experimental setup and the task itself before starting. For each participant data were collected over two sessions either over consecutive days or after a long break (~ 1 h).

### Stimulus and apparatus

#### Apparatus

Participants were seated in a double-walled sound attenuating booth (IAC, Winchester, UK), wearing headphones (Sennheiser HD 600), in front of a computer monitor and mouse. Sound was delivered through the headphones via a preamplifier connected to a signal generator (Tucker Davis Technology, RP2) which was in turn controlled by custom written software (MATLAB and TDT’s RPvdsEX). Participants registered responses to sounds via a custom designed graphical user interface (MATLAB) through mouse clicks at the end of each trial.

Sound levels were tested and calibrated using a condenser microphone (Bruel and Kjær, 4191) measuring amplifier (Bruel and Kjær 3110-003).

Sound stimuli were generated de novo for each trial using MATLAB and loaded onto the signal generator at the start of each trial. All sounds were generated and presented with a ~ 48 kHz sampling rate.

#### Signal

**Figure 1:**
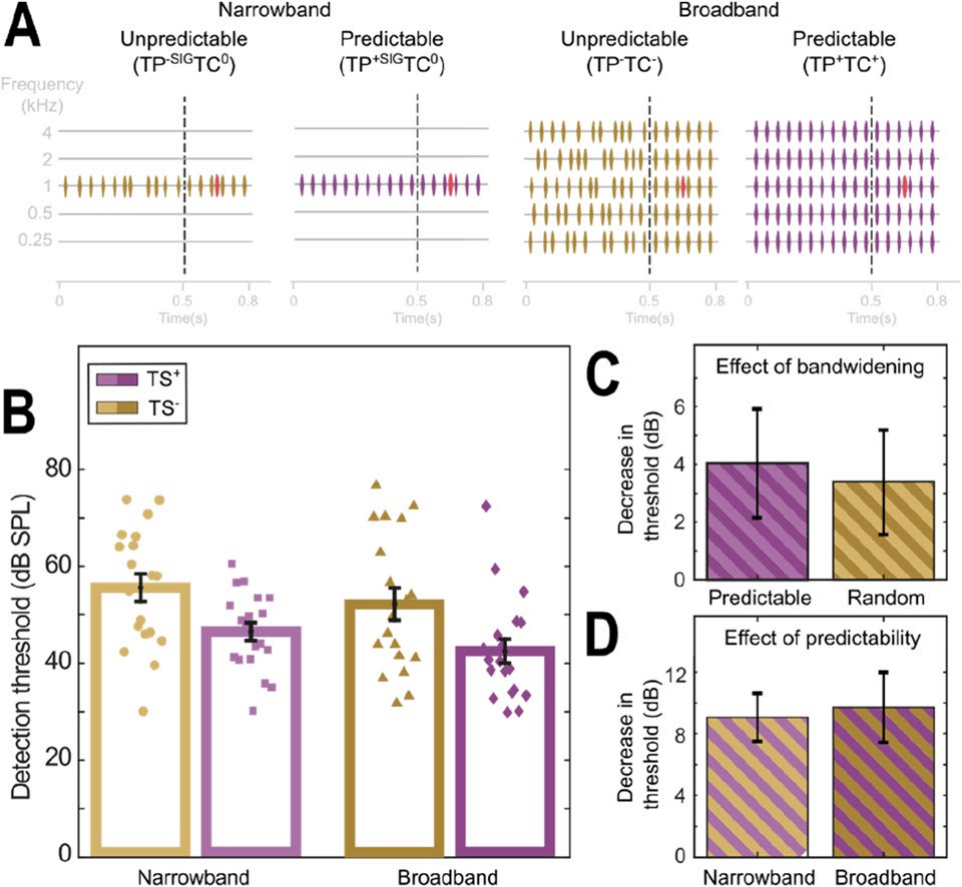
Temporal coherence and temporal predictability during build-up significantly affect subsequent detectability, when the two cues are not in conflict there is no evidence of an interaction. (**A**) Stimulus schematic. Each stimulus was composed of a background (purple/gold) and a pure tone signal (red). The backgrounds were a series of pure tone pips presented in either: (i) Left two panels) a narrowband (TC^0^ notation) or (ii) Right two panels) broadband (TP^+/−^ notation, 5 × 1 octave spaced tones centred at 1 kHz) configuration. Each background was composed of a pre-cursor (left of the vertical dashed line) and a masker (right of the vertical dashed line). The pre-cursor could either promote temporal predictability (purple and TP + notations, including TP^+SIG^) or disrupt temporal predictability (gold and TP^−^ notation, including TP^−SIG^). (**B**) Pure-tone detection thresholds for each condition in (**A**). The effect of bandwidening (**C**, in the predictable vs jittered conditions) and temporal predictability (**D**, in the narrowband vs broadband conditions) to detection thresholds. (**C**) The reduction in masking attributable to bandwidening was similar across the two temporal predictability conditions (Predictable = TP^+SIG^TC^0^ vs TP^+SIG^TC^0^ and Jittered = TP^−SIG^TC^0^ vs TP^-^TC^-^, Predictable =  ~ 4 dB, Jittered =  ~ 3.4 dB). (**D**) The reduction in masking attributable to adding temporal predictability (in the precursor) was also similar (Narrowband = TP^+SIG^TC^0^ vs TP^−SIG^TC^0^ and Broadband = TP^+^TC^+^ vs TP^−^TC^−^, Narrowband: ~ 9.1 dB, Broadband: ~ 9.7 dB).

Participants were required to detect a short (25 ms duration, 12 ms cos^2^ ramp) pure tone signal (1 kHz) that was embedded in a masker. Tone signals were presented in the trough between peaks 13 and 14 of the background (see below), meaning they were centred, temporally, in the middle of the masker (e.g. Fig. 1A). To measure tone detection thresholds, tones were varied in sound level according to an adaptive staircase rule (see procedure).

#### Background stimuli

To create the backgrounds, modulated pure tones (800 ms duration) were created each with a random carrier phase at the necessary frequencies for the narrowband (1 kHz, 65 dB SPL) and broadband conditions (1 kHz as before but also at: 0.25, 0.5, 2 and 4 kHz, termed ‘flanker bands’ where each was calibrated to be 65 dB SPL). The background was composed of two periods of time; the precursor (the first 500 ms) and the masker (the next 300 ms). In both experiments the masker was always temporally predictable (individual “pip” envelopes generated using ½ a cycle of a 40 Hz cosine which were then positioned at regular intervals at a 20 Hz rate). Temporal predictability, (TP), and temporal coherence (TC) in the precursor were varied in the two experiments. In Experiment 1 the precursor and masker could be broadband (TP^+^TC^+^ and TP^−^TC^−^, see Fig. 1) or narrowband (TP^+^TC^0^ and TP^−^TC^0^) where the precursor could be either 100% predictable (TP^+^TC^+^ and TP^+^TC^0^) or jittered (TP^−^TC^+^ and TP^−^TC^0^, each pip onset within the precursor was adjusted by a uniformly sampled random amount of time between the bounds ± 12.5 ms).

**Figure 2:**
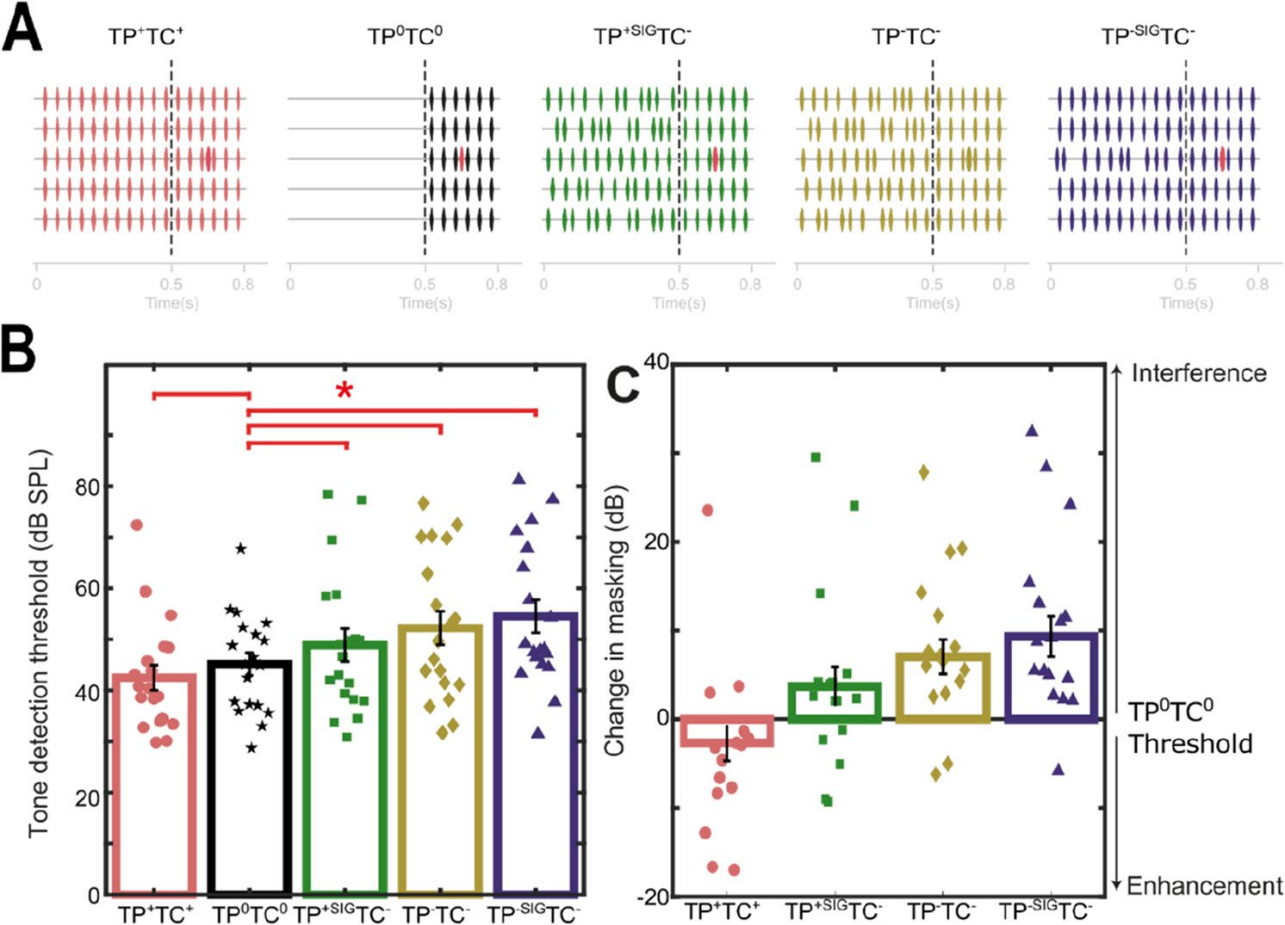
Discontinuity in sound statistics (temporal coherence and/or predictability) causes interference in the grouping process. (**A**) Stimulus schematic. Five conditions were contrasted: a neutral condition (i.e. no build-up: TP^0^TC^0^) and four where the build-up was varied (i.e. TP^+^TC^+^ = both temporal coherence and predictability, TP^+SIG^TC^−^ = Temporal predictability in the signal band only and without temporal coherence, TP^−^TP^−^ = Unpredictable (jittered) and no temporal coherence and TP^−SIG^TP^−^ = Temporal predictability and coherence in the flanking bands but a jittered signal band). (**B**) Tone detection thresholds for each condition. Red bars indicate significant differences between the neutral condition and the precursor conditions, identified in Table 2 (for full pairwise comparisons see Table 3) (**C**) The aim of these manipulations was to measure the relative contribution of “enhancement” (decrease in threshold w.r.t. the neutral condition) and “interference” (relative increase in threshold w.r.t. the neutral condition). Temporal predictability and coherence in the build-up tended toward enhancing the tone (though did not reach significance), whereas, jittering the signal band (TP^−SIG^TC^−^) or in all frequency bands (TP^−^TC^−^) produces significant interference.

In Experiment 2 the precursor and masker were always broadband and the temporal predictability and coherence of the signal band (1 kHz frequency band) and off-frequency components (0.25, 0.5, 2 and 4 kHz) were manipulated yielding 4 conditions with different precursors (see Fig. 2): temporally regular across all frequency bands (TP^+^TC^+^), jittered within the signal band (TP^−SIG^TC^−^), the flanker bands (TP^+SIG^TC^−^), or both (TP^−^TC^−^), and an additional ‘no-build up’, or neutral TP^0^, condition which just contained the masker with no precursor. Schematics of the stimuli in each experiment are included in Figs. 1A and 2A.

### Testing protocol

The experimental protocol was approved by the University College of London Research Ethics Committee and all research was performed in accordance with the relevant guidelines and regulations.

#### Staircase procedure

An adaptive staircase procedure was used to vary signal level between trials. A 3-down 1-up design was used with three sequential rules for varying the change in sound level (with rule changes being applied immediately after the last reversal): rule 1 = 3 reversals with a 6 dB step size, rule 2 = 3 reversals with a 2 dB step size and rule 3 = 4 reversals with a 1 dB step size. The tone signal level started at 65 dB SPL for each block.

#### General procedure

Tone detection threshold for each of the seven backgrounds were measured in blocks and 3 repeats of each threshold were measured. The intention was for that for each repeat, the order of the blocks would be re-randomised. Owing to an experimenter error this was only performed for 8 of the 19 participants. Nonetheless, statistical comparison of the mean thresholds across the three repeats for each condition for the two groups (i.e. randomised vs. same order participants) demonstrated there was no significant effect of the order within a repeat cycle between the two groups (paired t-test, uncorrected for multiple comparisons to add confidence, p > 0.05 for all conditions). Within each block a 3-interval forced choice design was used to probe tone signal detection. On a given trial 3 identical backgrounds, presented consecutively, were presented with a 1 s interval between each. Within one of these intervals a tone signal was added, and the participant was asked to identify the interval containing the tone using a mouse click on a response screen.

### Data analysis

Data was stored using MATLAB and converted into a suitable structures for analysis in SPSS. For each participant and testing block the threshold was calculated as the mean signal sound level across the final 4 reversals and we kept each of the 3 individual thresholds (i.e. 3 × repeats) for each condition. Due to relatively high across-participant variability and absence of exclusion criteria for poor performers we used Generalized Linear Mixed Models (GLMM, with a linear link function) to understand our data. Linear Mixed Models (LMMs) and Generalized Linear Mixed Models offer the opportunity to model random effects^34^, for example underlying differences in hearing ability, within the data and, hence, can reduce false positives and false negatives^35^. Hence, this was deemed an appropriate way to control for inter-individual differences in threshold. Analysis was performed in SPSS (IBM SPSS Statistics 27).

## Results

### Temporal predictability and temporal coherence can both independently influence grouping

To test the role of temporal predictability and temporal coherence in build-up we employed a simple tone in background stimulus design. In brief, participants (n = 19) were presented the same background 3 times in consecutive intervals (0.8 s duration, 1 s inter-stimulus interval), in one of these intervals a tone was also presented, and the participant had to identify this interval (i.e. 3-interval forced choice design). From trial-to-trial the sound level of a target tone was varied with a 3-down, 1-up adaptive staircase in blocks to yield a threshold for each block (the mean tone level across the last 4 reversals) with 3 repeats for each condition.

In Experiment 1, we compared the impact of manipulating temporal coherence and predictability in the precursor (Fig. 1A). To do this, four backgrounds were compared in a 2 × 2 design. The effect of across-channel temporal coherence was tested by altering the bandwidth, comparing a narrowband (1 kHz tone pips) and a broadband configuration (5 frequency complex between 0.25 and 4 kHz with 1 octave spacing). In addition, the effect of temporal predictability was tested by varying the stimulus statistics in the build-up (i.e. precursor) while maintaining identical stimulus statistics in the masker portion of the background, across comparisons (500–800 ms). The statistics in the masker period were always regular and were narrowband or broadband to match the build-up, but the temporal structure in each frequency channel during the build-up could either be regular (i.e. temporal predictability, Fig. 1A) or jittered (unpredictable, Fig. 1A). Note that for the broadband condition the temporally predictable configurations were also temporally coherent across frequency. In total Experiment 1 had four conditions: two narrowband conditions that allowed us to measure the effect of temporal predictability in the absence of across-channel temporal coherence, one condition was predictable during the build-up (TP^+SIG^TC^0^, Fig. 1A) and the other jittered (TP^−SIG^TC^0^). The two broadband conditions allowed the assessment of varying temporal predictability and coherence during build-up: one with temporal predictability and a temporally coherent build-up (TP^+^TC^+^) and one without (TP^−^TC^−^, though note that in both cases the masker did have temporal coherence and predictability).

Central to our hypothesis was the assumption that a lower (better) tone detection threshold would result when listeners were better able to segregate the masker from the competing tone. Comodulation Masking Release is a well-known auditory phenomena where adding temporally coherent off-frequency maskers paradoxically results in a lower thresholds^7^, thought to result from perceptual grouping of the signal and flanking channels^32,36–38^. We predicted that thresholds should be lowest in the TP^+^TC^+^ case, where the broadband masker would enable masking release and the build-up period contained a temporally predictable and temporally coherent structure matching that in the masker. We predicted that if temporal predictability plays a role in object formation both temporally predictable conditions should yield lower thresholds than the jittered conditions (i.e. TP^+^TC^+^ < TP^−^TC^−^ and TP^+SIG^TC^0^ < TP^−SIG^TC^0^).

Varying either bandwidth or temporal predictability had an effect on subsequent tone detection thresholds (Fig. 1B). Adding across-channel temporal coherence in the masker produced a moderate unmasking of the tone target, irrespective of temporal structure in the build-up (µ = 3.72 dB, SD = 7.67 dB). Altering the temporal structure, by transitioning from a jittered to a temporally regular modulation rate, whether broadband or narrowband (Fig. 1A), produced a large reduction (µ = 9.39 dB, SD = 7.13 dB) in tone detection thresholds. Tests revealed multicollinearity between predictors (Bandwidth, Predictability and Repeats) was negligible in these data (all LIF ≤ 1, all Pearsons, r ≪ 0.8). A Generalized Linear Mixed Model (GLMM, linear link function) was fitted with predictability, bandwidth, their interaction (predictability × bandwidth) and repeat as fixed factors and subject as the random factor (random intercept). A significant effect of both predictability (GLMM omnibus test of fixed effects: F(1, 222) = 71.990, p = 3.11 × 10^–15^) and bandwidth (fixed effects: F(1, 222) = 13.256, p = 3.38 × 10^–4^) was observed and this resulted in significant differences between levels in the model (see Table 1). There was no significant effect of repeats either across all levels (fixed effects: F(2, 222) = 0.429, p = 0.651) or between specific levels (Table 1). Improvements in threshold produced by unmasking due to bandwidth (Fig. 1C) and predictability (Fig. 1D) remained consistent across the other manipulation, confirmed by the non-significant significant interaction term (predictability × bandwidth: F(1, 222) = 0.091, p = 0.764, also see Table 1). The benefit observed by adding additional frequency bands was identical in magnitude for both jittered and predictable maskers, suggesting that varying temporal predictability during build-up did not disrupt the across-channel benefit of temporal coherence in the masker (Fig. 1C). In addition, the benefit of having temporal predictability within the precursor was also similar regardless of the bandwidth (Fig. 1D). Overall, predictability produced significantly larger reductions in threshold than band-widening (µ = 9.39 vs 3.72 dB, respectively, paired t-test, t(36) = 2.3598, p = 0.0238).

**Table 1:** Experiment 1: Fixed coefficients of the Generalized Linear Mixed Model (GLMM), including: estimate strength (Estimates), standard errors (Std. Errs), t-statistics (t), probability value (p-value) and 95% confidence intervals (Cis), for each fixed effect and the interaction bandwidth × predictability.

Parameter	Coefficient	Std. Err	t	p-value	95% CIs	
					Lower	Upper
Intercept	51.265	2.657	19.297	0	46.029	56.500
**Bandwidth**						
Narrowband (NB)	3.705	1.617	2.292	0.023	0.519	6.893
Broadband (BB)	Ref					
**Predictability**						
Unpredictable (UP)	− 9.751	1.754	− 5.559	7.718 × 10^8^	− 13.327	− 6.403
Predictable (P)	Ref					
**Repeat**						
Repeat 1	1.302	1.406	0.926	0.355	− 1.468	4.072
Repeat 2	0.662	1.293	0.512	0.609	− 1.886	3.211
Repeat 3	Ref					
**Bandwidth × predictability**						
NB × UP	0.672	2.234	0.301	0.764	− 3.729	5.074
NB × P						
BB × UP						
BB × P	Ref					

### Build-up critically influences the subsequent use of both temporal predictability and coherence cues

In Experiment 1 it was difficult to tease apart the relative contribution of temporal predictability and temporal coherence to subsequent perception. For example, providing predictability and coherence in the build-up (TP^+^TC^+^) could facilitate grouping of the on-frequency channel into the background and, hence, enhance tone detection. Alternatively, disrupting predictability and coherence during the build-up (TP^−^TC^−^) could interfere with subsequent grouping and tone detection. When comparing these conditions, and observing a difference, it is unclear whether we are measuring either one or both. To allow quantification of the relative enhancement/interference on subsequent performance we introduced a “neutral” condition where the build-up period had been removed (Fig. 2A, TP^0^TC^0^). This allowed us to determine the relative direction of change in performance that was elicited by the inclusion of each respective precursor.

Experiment 1 suggests that temporal predictability and coherence are independent processes, as the size of one was not significantly varied when changing the parameters of the other (and confirmed by the non-significant interaction term in the GLMM). However, in this experiment the jittered precursor transitioned to a regular sequence of tones in the masker. These conditions favour treating the precursor and masker as independent from one another, as this transition is likely to be interpreted as the start of a new auditory object. In contrast in the predictable case the continuity in statistics will cause the precursor and masker to be grouped together. For the broadband case the cues are consistent across all frequencies. We next sought to test how temporal coherence and predictability cues interact when put into conflict (from precursor to masker), by independently modulating the temporal structure of the flanking and signal bands in the build-up period.

In Experiment 2 we generated two additional conditions, each designed to promote different grouping configurations, while disrupting the grouping of flanking and signal bands during the precursor. The first promoted the use of temporal predictability to group just the signal band, by providing continuous regularity in the signal channel from precursor to masker, while jittering the timing of the flanking bands in the precursor (TP^+SIG^TC^−^, Fig. 2A). This manipulation aimed to block the use of temporal coherence in the masker but allow the use of temporal predictability in the signal band. The second sought to block both the use of temporal coherence (across-channels) and temporal predictability (within the signal band, TP^−SIG^TC^−^).

A significant effect of varying the masker properties during build-up was found across the 5 precursor conditions (Generalized Linear Mixed Model with linear link function, Fixed effects: predictability and repeat Random effects: subject, n = 19, F(4, 278) = 18.002, p = 3.688 × 10^–13^, and no effect of repeat: F(2, 278) = 1.851, p = 0.159). Tone detection thresholds were lowest when the build-up contained identical sound statistics to the masker period (TP^+^TC^+^, µ = 42.5 dB SPL) suggesting an enhancement relative to the neutral condition (TP^0^TC^0^, µ = 45.12 dB SPL, 2.62 dB difference). This was confirmed by the significant negative coefficient for TP^+^TC^+^, referenced to TP^0^TC^0^ (Table 2). However, this effect was relatively small (coefficient = − 3.076). By contrast, conditions with jitter in the build-up produced interference, relative to the neutral condition (Fig. 2B: TP^−^TC^−^, µ = 52.21, TP^−SIG^TC^–^, µ = 54.5, and TP^+SIG^TC^−^ µ = 48.89 dB SPL, or 7.1, 9.38 and 3.77 dB increases respectively). Model coefficients revealed interference was significant across all 3 of the interference conditions, relative to the neutral condition (see Table 2).

**Table 2:** Experiment 2: Fixed coefficients of the Generalized Linear Mixed Model (GLMM), including: estimate strength (Estimates), standard errors (Std. Errs), t-statistics (t), probability value (p-value) and 95% confidence intervals (Cis), for each fixed effect and the interaction bandwidth × predictability.

Parameter	Coefficient	Std. Err	t	p-value	95% CIs	
					Lower	Upper
Intercept	51.265	2.657	19.297	0	46.029	56.500
**Pre-cursor**						
TP^+^TC^+^	− 3.076	1.4806	− 2.078	0.039	− 5.991	− 0.162
TP^0^TC^0^	Ref					
TP^+SIG^TC^−^	3.547	1.398	2.537	0.012	0.794	6.299
TP^+^TC^+^	6.432	1.369	4.697	0.000004	3.736	9.128
TP^−SIG^TC^−^	9.238	1.554	5.946	8.223 × 10^–9^	6.180	12.297
**Repeat**						
Repeat 1	2.121	1.175	1.805	0.072	− 0.193	4.435
Repeat 2	0.518	1.216	0.426	0.670	− 1.876	2.913
Repeat 3	Ref					

Pairwise comparisons were used to reveal additional differences between conditions (see Table 3). Given the significant difference found between the TP^+^TC^+^ and TP^0^TC^0^ condition it is not surprising that TP^+^TC^+^ was significantly lower than all other conditions (β = − 5.97, − 8.54 and − 11.58, t(278) = − 4.586, − 6.380 and − 6.787, for TP^+SIG^TC^−^, TP^−SIG^TC^−^ and TP^−^TC^−^, see Table 3 for p-values). Neither TP^+SIG^TC^−^ and TP^−SIG^TC^−^ were significantly different from TP^−^TC^−^ (TP^+SIG^TC^−^ vs TP^−^TC^−^: β = − 2.565, t(278) = 2.412, p = 0.058, TP^−SIG^TC^−^ vs TP^−^TC^−^: β = 3.018, t(278) = 1.489, p = 0.058) but were significantly different from one another (TP^+SIG^TC^−^ vs TP^−SIG^TC^−^: β = − 5.583, t(278) = 0.02). Together these results demonstrate that the preceding sound statistics can significantly affect subsequent sound in noise detection. Interference is largest when the signal band’s temporal statistics are unpredictable (i.e. TP^−^TC^−^ and TP^−SIG^TC conditions). Smaller changes are observed when temporal predictability in the build-up is present (TP^+SIG^TC^−^, i.e. when only disrupting temporal coherence in the flanking bands) or providing both cues (TP^+^TC^+^).

**Table 3:** Experiment 2: Sequential Bonferonni adjusted probability values for pairwise comparisons.

	TP^+^TC^+^	TP^0^TC^0^	TP^+SIG^TC^−^	TP^−SIG^TC^−^	TP^−^TC^−^
TP^+^TC^+^	1	0.036643	4.78E−05	6.62E−09	6.90E−10
TP^0^TC^0^	0.036643	1	0.04954	4.78E−05	1.16E−06
TP^+SIG^TC^−^	4.78E−05	0.04954	1	0.058167	0.002033783
TP^−SIG^TC^−^	6.62E−09	4.78E−05	0.058167	1	0.058166818
TP^−^TC^−^	6.90E−10	1.00E−06	0.002034	0.058167	1

### Build-up can be used to block the subsequent use of temporal coherence cues

In Experiment 2, conditions that discouraged grouping of the signal and flanking bands during build-up, i.e. TP^+SIG^TC^−^ and TP^−SIG^TC^−^, appeared to produce comparable thresholds to those observed in the narrowband conditions in Experiment 1 (Fig. 1B). As the same subjects completed Experiment 1 and 2 we were able to directly compare thresholds across both. Therefore, thresholds for the narrowband conditions in Experiment 1 (TP^+^TC^0^ and TP^−^TC^0^) were directly compared to the relevant conditions in Experiment 2 (TP^+SIG^TC^−^ and TP^−SIG^TC^−^, respectively). We fit our data with a GLMM (linear link function) with bandwidth, jitter and their interaction as fixed factors and subject as the random factor (random intercepts). This yielded a significant effect of predictability (F(1, 224) = 43.860, p 2.582 × 10^–10^, p < 0.05 after Bonferonni correction for this additional test, also see Table 4 for Fixed Coefficients) but not of bandwidth (F(1, 224) = 0.322, p = 0.571). In addition, no interaction was found (predictability × bandwidth, F(1, 223) = 2.816, p = 0.095), although the β coefficient was not negligible (β = 3.656, see Table 4). These results suggest that the benefits of bandwidening observed in Experiment 1 (Fig. 1C) could be blocked by the addition of incoherent flanking/signal band envelopes in the build-up period.

**Table 4:** Fixed coefficients of the Generalized Linear Mixed Model (GLMM), including: estimate strength (Estimates), standard errors (Std. Errs), t-statistics (t), probability value (p-value) and 95% confidence intervals (Cis), for each fixed effect and the interaction bandwidth × predictability.

Parameter	Coefficient	Std. Err	t	p-value	95% CIs	
					Lower	Upper
Intercept	54.399	2.721	19.991	0	49.037	59.761
**Bandwidth**						
Narrowband (NB)	1.21	1.607	0.753	0.452	1.958	4.378
Broadband (BB)	Ref					
**Predictability**						
Unpredictable (UP)	− 5.387	1.742	− 3.092	0.002	− 8.819	− 1.953
Predictable (P)	Ref					
**Bandwidth × predictability**						
NB × UP	− 3.656	2.178	− 1.678	0.095	− 7.949	0.637
NB × P						
BB × UP						
BB × P	Ref					

## Discussion

We employed a simple paradigm to assess how manipulating grouping cues during ‘build-up’ influenced subsequent sound segregation, as assessed by a tone detection task. We found that those grouping cues available during build-up critically shaped subsequent perception. Our design allowed us to assess whether the build-up period produced enhancement (an improvement in tone detection threshold) or interference (a reduction in tone detection threshold). The addition of a temporal predictable/coherent preceding sound produced a small, but significant, enhancement. Conversely, adding a period of jitter during build-up (whether: at/away from the signal frequency or across all frequencies) produced significant interference. In addition, interference was significantly larger when the signal band was jittered versus when the flanking bands were jittered. Finally, disrupting temporal coherence between signal and flanker bands during the build-up also produced significant increases in thresholds. Thus, these results demonstrate the influence of early sound statistics on subsequent sound perception.

As mentioned already, in Experiment 1 we found no evidence of the build-up period promoting or interfering with the ability to benefit from temporal coherence in the masker. Maintaining temporal predictability in the signal band while disrupting temporal coherence (TP^+SIG^TC^−^) produced significant interference, however, the thresholds were similar to those of the narrowband condition (Experiment 3). This suggests, though does not prove, that this interference was most likely the loss (or blocking) of the temporal coherence advantage, as described previously^39–41^. This is not “true” interference of temporal coherence (i.e. a decrease in threshold relative to a neutral condition) but rather a loss of enhancement. This suggests that either interference/enhancement due to build-up has a different time integration window for temporal coherence than for temporal predictability, which may have a shorter time constant. Frequently in the study of temporal coherence, particularly in streaming, the temporally coherent stimuli are also temporally predictable (during build-up and thereafter), meaning it possesses both temporal predictability and temporal coherence. Our data highlight the need to separate these two cues when studying temporal coherence.

The general pattern of results observed here is consistent with the idea that object formation during build-up is influential to subsequent perception, at least within the relatively recent context (hundreds of millisecond scale). Within our results we observed both enhancement and interference, though it is a complex task to understand the contribution of each cue. Previous work demonstrated that surrounding a period of temporal coherence with random temporal structure (similar to TP^−^TC^−^, but with a post-cursor) disrupts the benefits of temporal coherence^39,40^. Grose et al.^40^ presented 4 continuous narrow bands of noise (20 Hz wide centred at 804, 1200, 1747 and 2503 Hz) with a temporal structure that was switched from being comodulated (i.e. temporally coherent) to randomly modulated, while trained participants were asked to detect pure tones. The authors found that incoherently modulated precursors/postcursors (both were modified in each trial) produced a small interference with comodulation masking release, elevating thresholds by ~ 2 dB. Subsequent work by, largely, the same authors^39^ demonstrated a similar effect in highly trained participants (n = 4), though with a much larger effect size (~ 10 dB). Similarly, a large interference (9.55 dB) was observed in our study when replacing a comodulated build-up with a temporally jittered one (n = 19, minimal training provided). Our work, which only looked at the influence of precursor sounds, also demonstrated an interference attributable to disruption of temporal predictability in the build-up (Fig. 1D), suggesting the interference observed by Grohse and colleagues is largely attributable to build-up rather than postcursor interference. It is also worth noting that this study used a harmonic pure tone complex, rather than ERB spaced noise bands. It is possible these results only hold for harmonic complexes, an across-frequency grouping cue. However, the similarity of the results here, with the Grohse study, suggests that using a harmonic complex is not critical. In addition, changes in temporal predictability were similar in both narrowband and broadband conditions in Experiment 1 (Fig. 1D), though further research would be needed to investigate this relationship.

In the present study, conditions that discouraged grouping of the signal and flanking bands, i.e. TP^+SIG^TC^−^ and TP^−SIG^TC^−^, produced thresholds comparable to those observed in the narrowband conditions. This suggests that build-up could be used to block the subsequent use of temporal coherence cues (see the last paragraph of the results), consistent with previous findings (see above). However, in Experiment 1 the temporal coherence benefit was comparable for the predictable and unpredictable comparisons (see Fig. 1C). Here, the lack of interaction demonstrates that bandwidening produced the similar effects regardless of the coupling (TC^+^)/decoupling (TC^−^) of channels during build-up. This dichotomy of results has interesting implications. Temporal incoherence, in the pre-cursor, was not sufficient to block the benefit of bandwidening, i.e. the Comodulation Masking Release (Experiment 1). However, when temporal predictability was available in one or more channels (i.e. TP^+SIG^TC^−^ and TP^−SIG^TC^−^) thresholds were similar to narrowband conditions (suggesting across channel processing was blocked and the benefit of temporal coherence lost). If true, these results would suggest, along with previous studies, that continuity (of temporal predictability) can block the use of temporal coherence, which might have implications for our understanding of the temporal coherence model, i.e. “that stream formation depends primarily on temporal coherence between responses that encode various features of a sound source”^4^. However, it is critical to note that absence of proof is not proof of absence, i.e. demonstrating no statistical difference exists does not prove there was no difference. Hence, further testing would be required to explore this possibility.

It should be noted that the masker period had a very short build-up (between the offset of the pre-cursor and the onset of the tone) of 187.5 ms. It may be that the large change in sound statistics is sufficient to “reset” build-up, similar to resetting of stream segregation^25,26^, and there is time for temporal coherence to build-up and allow the unmasking observed in Experiment 1, with no additional enhancement or interference due to jitter/predictability in the precursor. Previous studies have suggested that unmasking due to precursors is initially a “temporal decline in masking” over the first ~ 400 ms (also observed for sound with no temporal coherence) and then a large threshold decrease (in a subset of participants) after 400 ms^41,42^. If there is a build-up benefit to temporal coherence then there was not sufficient time in this experiment for it to manifest (in just the masker) and, hence, it is unlikely we observed an additional enhancement from build-up in the masker, beyond a temporal decline in masking.

The variability of thresholds across individuals observed in this study was relatively large (e.g. see Fig. 1), this could have been for a number of reasons. Firstly, we chose to use a small number of reversals for our final rule (4 reversals). We were concerned about increasing the duration of the task (by adding extra reversals) as it is already an attentionally demanding task and the length of time it takes to complete the study is quite long (> 3 h on average, usually over 2 days). Secondly, studies on this topic (and more generally) frequently use an exclusion criteria, e.g. any participant whose thresholds were above a criterion of variance would be excluded from the data set. This approach can significantly reduce inter-individual variability but also means it’s difficult to know how representative the results are with respect to the general population. Finally, participants were only provided with a small amount of training—roughly 15-20 min. A greater duration of training or a requirement to reach an absolute threshold level during training can also be used to exclude those who perform poorly on the task. Again, this reduces variability but also means it is difficult to know how representative the data are of the general population. In the future changes could be made to reduce variance though careful consideration would need to be taken on understanding how this would influence sampling and how much it would extend the duration of the experiments.

In conclusion, this work supports the idea that build-up is a critical process for shaping subsequent perception. Manipulating temporal coherence and predictability during build-up can produce independent changes to subsequent tone detection (Experiment 1). In addition, manipulating the preceding sound can produce both enhancement and interference in subsequent sound detection. This study lays the foundation for future work that could tease apart the contributions of temporal grouping cues and improve our understanding of the interaction between grouping cues more generally.

## Data Availability

The dataset from the current study is publicly available through Figshare: https://figshare.com/s/619b2bb486a4d226c3b3.
